# The Evidence for Increased L1 Activity in the Site of Human Adult Brain Neurogenesis

**DOI:** 10.1371/journal.pone.0117854

**Published:** 2015-02-17

**Authors:** Alexey A. Kurnosov, Svetlana V. Ustyugova, Vadim I. Nazarov, Anastasia A. Minervina, Alexander Yu. Komkov, Mikhail Shugay, Mikhail V. Pogorelyy, Konstantin V. Khodosevich, Ilgar Z. Mamedov, Yuri B. Lebedev

**Affiliations:** 1 Laboratory of Comparative and Functional Genomics, Shemyakin-Ovchinnikov Institute of Bioorganic Chemistry Russ. Acad. of Sci., Moscow, Russia; 2 Division of Biology and Biological Engineering, California Institute of Technology, Pasadena, California, United States of America; 3 Department of Information Technology and Automated Systems, National Research University Higher School of Economics, Moscow, Russia; 4 Department of Clinical Neurobiology, Heidelberg University Hospital at German Cancer Research Center (DKFZ), Heidelberg, Germany; University of Nebraska Medical Center, UNITED STATES

## Abstract

Retroelement activity is a common source of polymorphisms in human genome. The mechanism whereby retroelements contribute to the intraindividual genetic heterogeneity by inserting into the DNA of somatic cells is gaining increasing attention. Brain tissues are suspected to accumulate genetic heterogeneity as a result of the retroelements somatic activity. This study aims to expand our understanding of the role retroelements play in generating somatic mosaicism of neural tissues. Whole-genome Alu and L1 profiling of genomic DNA extracted from the cerebellum, frontal cortex, subventricular zone, dentate gyrus, and the myocardium revealed hundreds of somatic insertions in each of the analyzed tissues. Interestingly, the highest concentration of such insertions was detected in the dentate gyrus—the hotspot of adult neurogenesis. Insertions of retroelements and their activity could produce genetically diverse neuronal subsets, which can be involved in hippocampal-dependent learning and memory.

## Introduction

Approximately 40% of the human genome is comprised of multiple copies of retroelements (REs) due to their winning streak in the course of mammalian evolution [1]. The major groups of retroelements present in the human genome are the Long Terminal Repeats (LTR) retrotransposons, the Long Interspersed Nuclear Elements (LINEs), the Short Interspersed Nuclear Elements (SINEs) (most of which belong to the Alu family), and the SINE-R/VNTR/Alu (SVA) elements. The only family of autonomous non-LTR REs known to be currently active is L1. The mobility of non-LTR REs from the other two groups (Alu and SVA) relies on the L1 retrotransposition machinery. Polymorphic insertions found in the genome of a part of human population usually result from the recent retrotranspositional activity of REs which have retained the capacity to amplify themselves. These insertions belong to the evolutionary young RE groups. Around 60–80 insertions of LINE elements in the human genome considered to be capable of retrotransposing belong to the L1Hs subfamily [2]. Most of the active Alu copies are attributed to the AluYa5 and AluYb8 subfamilies [3–5]. Insertions of retroelements can lead to reshaping of the genome structure and alteration of nearby genes expression [6, 7]. Some of such insertions have been reported to cause various diseases [8, 9].

It has been long considered that the majority of retrotranspositions occur in the germ line, while the mobility of REs in somatic cells is strictly suppressed. Mammalian cells possess multiple mechanisms of inhibiting REs expression, including siRNA [10], miRNA [11], piRNA-induced L1 methylation [12], and repression of the methylated L1 promoters by methyl-CpG-binding protein 2 (MeCP2) [13]. Recently, however, sufficient data have been accumulated suggesting that the REs activity may be a common property of cells in somatic tissues [14, 15]. Thus, somatic L1 and Alu insertions have been found in cancerous cells [16–19]. Furthermore, studies with the retrotransposition-reporter L1-EGFP plasmid in human embryonic stem cell lines [20] and the rat and mouse models [21] provided evidence for mobilization of L1 retroelements in embryonic tissues. An increased frequency of retrotransposition events in neurogenesis was demonstrated by introducing the L1-retroposition-reporter construct into the rat [22] and human [23] neural progenitor cells. An increase in the number of L1 copies in the human brain tissues in comparisons with non-neural tissues was found by quantitative PCR (qPCR) [23]. Somatic REs insertions were also detected in the human brain and induced pluripotent stem cells by next generation sequencing of the DNA libraries enriched in RE-containing sequences [24, 25]. This approach allowed to reveal thousands of somatic L1, Alu, and SVA integrations in the hippocampi and caudate nuclei of the donors. However, whole-genome L1 profiling in single neurons from the human caudate nucleus and cerebral cortex assessed the frequency of the somatic insertions at less than 0.6 L1 insertions per neuron [26]. Whole-genome sequencing was also applied to identify somatic L1 retrotranspositions in the brains of patients with schizophrenia where an increased number of mobile elements insertions was predicted by qPCR analysis [27]

Here we applied a high-throughput approach to directly compare the number of autonomous (L1) and non-autonomous (Alu) retroelement somatic insertions in various human adult brain regions and a control non-nervous tissue. Whole-genome Alu and L1 profiling was performed for four brain regions including the dentate gyrus, the only region of the adult human brain that was shown to retain significant neurogenic capacity.

## Results

### Sample choice: neurogenic and non-neurogenic tissues

Previously, activation of L1 retrotranspositions was associated with a switch from neural stem cells to fast proliferating neural progenitor cells *in vitro* and *in vivo* in the mouse dentate gyrus [22, 28]. The subgranular zone (SGZ) of the dentate gyrus (DG) together with the subventricular zone (SVZ) of the lateral ventricles are the only regions in the mammalian brain that persist in generating new neurons throughout the animal life [29]. In the adult human brain, substantial neurogenesis was described in the dentate gyrus [30, 31], whereas the data regarding SVZ neurogenesis is controversial [32–35]. To analyze whether continuous proliferation of precursor cells affects somatic retrotranspositions, we chose five samples taken from different tissues of a single individual for the whole-genome L1 and Alu profiling. The tissues represented neurogenic brain regions (the DG and the potentially neurogenic SVZ), non-neurogenic brain regions (the cerebellum and the frontal cortex), and a non-neural control tissue (the myocardium).

### Retrieving somatic insertions by next generation sequencing

**Fig 1:**
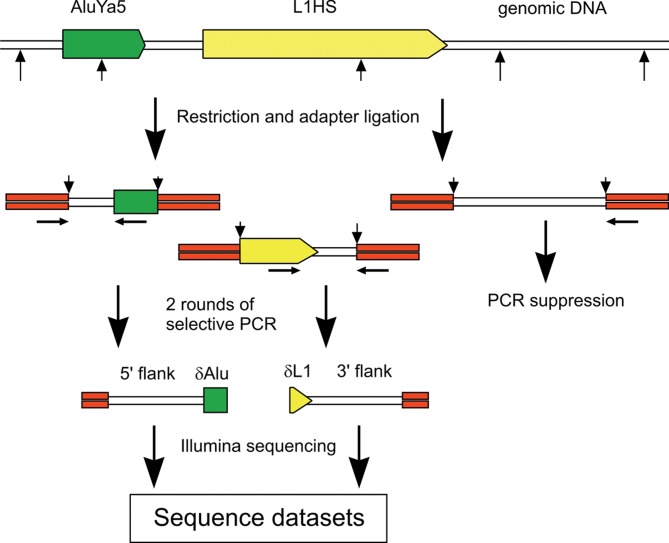
Retroelements flanking sequences library preparation. Small vertical arrows show the restriction sites. Horizontal arrows show PCR primers.

For library preparation, we modified the suppression PCR-based method of retrieving REs terminal and flanking sequences from genomic DNA [36, 37]. Briefly, our protocol of somatic insertion identification included the following steps (Fig. 1; see Experimental Procedures for more details): 1) Ligation of the suppression adapters to the restricted genomic DNA. 2) Two steps of suppression PCR selectively amplifying REs of the AluYa5 or the L1Hs subfamily. L1 libraries were constructed from the L1 3’-termini as the LINEs often appear 5’-truncated. On the contrary, we have chosen 5’-flanking sequences of the Alu repeats for the construction of the libraries in order not to include 3’-polyA-sequences of the Alu into the libraries and thus to spare the informative reads length. DNA molecules in the produced libraries consisted of a short retroelement fragment, its flanking sequence, which served to identify an insertion coordinate, and an adaptor sequence. The libraries were comprised of the DNA fragments representing two types of insertions: fixed and germline insertions (coming from all cells of the samples) and somatic insertions (coming from few or even one cell). 3) Illumina sequencing of the DNA libraries. 4) Mapping of the reads to the reference human genome and analysis of the mapping results which aimed to reveal the coordinates of potentially somatic insertions. We defined an insertion as potentially somatic if its coordinate did not match the known REs insertion coordinates in the reference genome and if it belonged to the library originating from only one of the studied tissue samples. Another constraint imposed on the reads potentially representing somatic retrotranspositions was the possibility to establish the insertion coordinate with a single-base resolution. Thus, only the pairs of the Illumina reads which had a mapping-informative part in the retroelement-containing read could be considered to represent potentially somatic insertions.

A total of 10,709,681 and 61,213,133 high-quality reads were obtained for L1 and Alu libraries, respectively. The detailed information on the number of reads and somatic insertions detected in each library is shown in Table 1. 817 out of 1,528 (53.5%) reference L1Hs and 2,933 out of 3,918 (74.9%) reference AluYa5 insertions were detected in the libraries. We have also found the coordinates of 167 non-reference L1 and 653 non-reference Alu retroelements which were detected in all tissues and therefore can be regarded as polymorphic or novel germline insertions.

**Table 1:** The number of potentially somatic L1 and Alu insertions detected in different tissue samples and the data on their distribution in genome.

	cerebellum	frontal cortex	SVZ	DG	myocardium
**L1**					
**Number of high-quality reads**	2723127	825363	1845367	3435529	1880295
**Number of reads representing potentially somatic insertions**	1712	475	1161	3211	1170
**Number of potentially somatic insertions**	1651	462	1133	3100	1151
**% of reads representing potentially somatic insertions**	0.0629	0.0576	0.0629	0.0935	0.0622
**Number (%) of somatic L1 detected in genes**	842 (51.00)	236 (51.08)	584 (51.54)	1558 (50.26)	578 (50.22)
**Number (%) of somatic L1 detected in 5 kB upstream genes**	92 (5.57)	31 (6.71)	74 (6.53)	177 (5.71)	62 (5.39)
**Alu**					
**Number of high-quality reads**	11978540	11962901	10921385	13339041	13011266
**Number of reads representing potentially somatic insertions**	1376	2217	1353	3079	1275
**Number of potentially somatic insertions**	1317	2138	1308	2984	1243
**% of reads representing potentially somatic insertions**	0.0115	0.0185	0.0124	0.0231	0.0098
**Number (%) of somatic Alu detected in genes**	623 (47.30)	1028 (48.08)	609 (46.56)	1465 (49.10)	589 (47.39)
**Number (%) of somatic Alu detected in 5 kB upstream genes**	67 (5.09)	105 (4.91)	61 (4.66)	105 (3.52)	55 (4.42)
**Combined set of L1 and Alu**					
**% of somatic retroelements detected in genes**	49.36	48.62	48.87	49.69	48.75
**% of somatic retroelements detected in 5 kB upstream genes**	5.36	5.23	5.53	4.64	4.89

**Fig 2:**
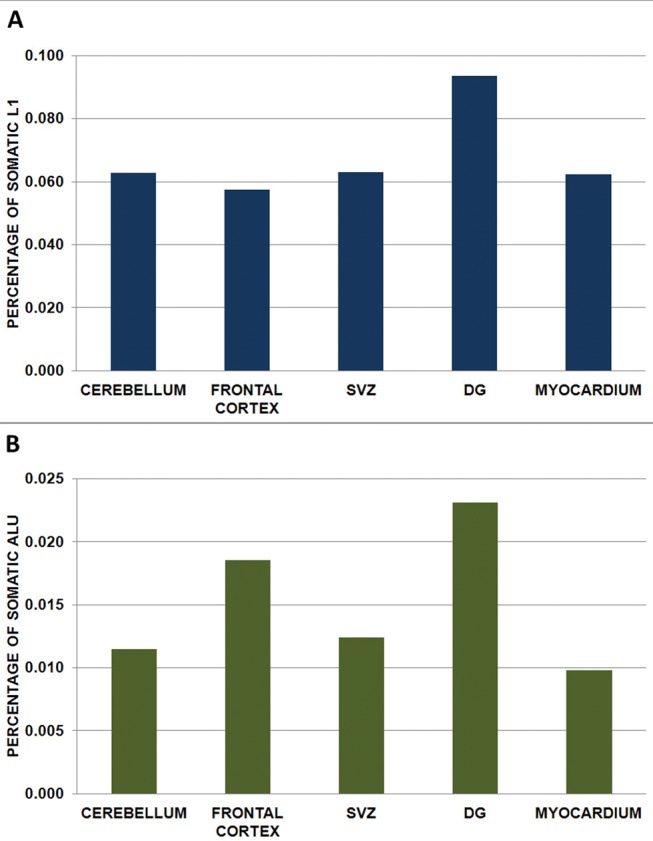
Normalized number of the somatic insertions (number of reads representing somatic insertions divided by the total number of reads) in the DNA of the studied samples. (*A*)—L1 insertions; (*B*)—Alu insertions. See also Table 1.

A total of 7,497 potentially somatic L1 and 8,990 potentially somatic Alu insertions were identified in the tissue libraries (Table 1). All the detected insertions were represented by a very low number of reads (not more than by 4 reads for L1 and 5 for Alu, though overwhelmingly by one read). As the number of detected somatic insertions obviously depends on the total number of reads in a library, we normalized the number of insertions by dividing it by the number of reads. The percentage of the reads derived from potentially somatic insertions differed among the libraries (see Fig. 2). Strikingly, whereas the percentage of somatic L1 insertions was approximately equal for the cerebellum, cortex, SVZ and myocardium (0.058–0.063%), the dentate gyrus cells exhibited significantly higher percentage of L1 retrotranspositional events (0.093%) (Fig. 2A; p<0.0001, Poisson test). Pair-wise Poisson tests also confirmed that only the dentate gyrus exhibited a higher rate of L1 integration compared to other brain regions and myocardium, with the latter not being different from each other (p<0.0001 for the dentate gyrus and p>0.05 for all other samples, Poisson tests). The highest percentage of somatic Alu insertions (0.023%) was observed in the DG (Fig. 2B). However, in contrast to L1 data, the frontal cortex also exhibited a comparatively high percentage of somatic Alu insertions (0.018%). The percentage of somatic Alu insertions for the cerebellum, SVZ, and myocardium was much lower (0.011, 0.012 and 0.010%). Nevertheless, using the pair-wise Poisson tests we found that not only the dentate gyrus and the frontal cortex, but also the cerebellum, SVZ, and myocardium samples were different from all the other samples (except cerebellum vs SVZ, where p = 0.0506). Thus, whereas somatic L1 insertions were equally distributed in the analyzed samples (~0.06%), with the exception of the dentate gyrus, the percentage of somatic Alu insertions varied among different brain regions.

**Fig 3:**
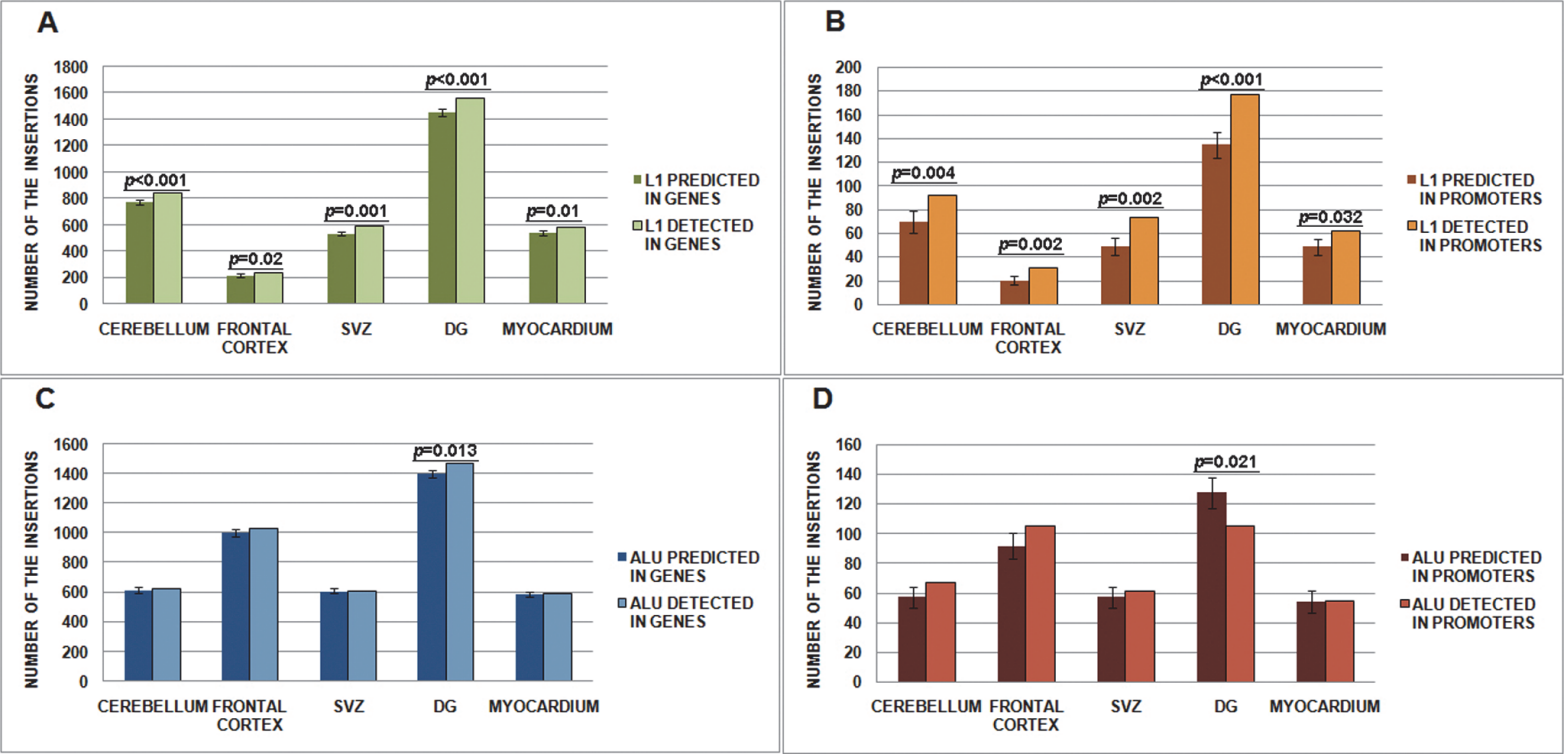
Number of the retroelement insertions detected within genes and promoters (for each library, predictions are derived from 1000 simulations of coordinates sample sets). Error bars show 1 SD. (A)—L1 in genes; (B)—L1 in promoters; (C)—Alu in genes; (D)—Alu in promoters.

A total of 3,798 somatic L1 and 4,314 somatic Alu elements from all libraries integrated into genes (overwhelmingly into introns). Another portion of somatic insertions (436 L1 and 393 Alu) was detected within 5,000 bp upstream of the gene transcription start sites (Table 1). In order to test whether the distribution of the detected insertions in the genome was random, we generated simulated coordinate datasets of the sizes identical to the sizes of the experimental datasets. Repeating the simulation 1000 times for each set gave ranges of the number of insertions, which were expected to be found in genes or promoter regions. These ranges were compared to our experimental data. In all brain regions, the number of L1 insertions was significantly higher than predicted both in genes (with the p-values < 0.001; = 0.02; = 0.001; < 0.001; = 0.01 for the cerebellum, the frontal cortex, the SVZ, the DG, and the myocardium, respectively; Monte-Carlo test, 1000 permutations) and promoters (with the p-values = 0.004; = 0.002; = 0.002; < 0.001; = 0.032 for the cerebellum, the frontal cortex, the SVZ, the DG, and the myocardium, respectively; Monte-Carlo test, 1000 permutations) (Fig. 3A and 3B). The Alu insertion rate matched the predicted rate in genes for all samples except the DG (Fig. 3C), where the number of Alu was significantly higher than predicted (p = 0.013; Monte-Carlo test, 1000 permutations). Additionally, the DG was the only tissue which demonstrated the lower than predicted Alu insertion rate in promoter regions (p = 0.021; Monte-Carlo test, 1000 permutations) (Fig. 3D).

Interestingly, across all samples analyzed, the distribution of somatic L1 or Alu insertions was very similar, namely, the percentages of somatic L1 or Alu that integrated into the genes or 5 kb region upstream genes were 50–51% and 5.5–6.5% for L1 or 47–49% and 3.5–5% for Alu, respectively (Table 1). The same held true for the combined set of somatic L1+Alu (Table 1). The lack of variability was confirmed by an overdispersion test (in all cases p > 0.98).

Finally, we analyzed the orientation of somatic retroelement insertions relative to genes (for those retroelements that integrated into introns or 5 kb regions upstream genes). Strikingly, intronic L1 elements preferentially integrated in the opposite orientation relative to the gene (Table 2) −40.96 ± 0.31% (mean±SD) of L1 were co-oriented; 59.05 ± 0.29% (mean±SD) of L1 were counter-oriented (analysis of distribution across different brain regions and myocardium: p = 0.9999, overdispersion test; analysis of distribution in a single brain region or myocardium: p < 0.0001 for all samples, binomial test, the null hypothesis was no preference in orientation). We observed no preference in the orientation of L1 integrations in the promoter regions (binomial test). The integration of Alu in both promoter and intronic regions also exhibited no preference in orientation (p > 0.05), with the exception of Alu in the dentate gyrus, (p = 0.032, binomial test).

**Table 2:** The orientation of somatic L1 and Alu insertions relative to nearby genes.

	cerebellum	frontal cortex	SVZ	DG	myocardium
**L1**					
**Number of somatic L1 detected in genes** (p-value*)	842 (<0.001)	236 (0.02)	584 (0.001)	1558 (<0.001)	578 (0.01)
Number (%) of CO-oriented somatic L1	345 (40.97)	97 (41.10)	239 (40.92)	644 (41.34)	234 (40.48)
Number (%) of COUNTER-oriented somatic L1	497 (59.03)	139 (58.90)	345 (59.08)	914 (58.66)	344 (59.52)
**Number of somatic L1 detected in 5 kB upstream gene** (p-value*)	92 (0.004)	31 (0.002)	74 (0.002)	177 (<0.001)	62 (0.032)
Number (%) of CO-oriented somatic L1	38 (41.30)	20 (64.52)	31 (41.89)	88 (49.72)	34 (54.84)
Number (%) of COUNTER-oriented somatic L1	54 (58.70)	11 (35.48)	43 (58.11)	89 (50.28)	28 (45.16)
**Alu**					
**Number of somatic Alu detected in genes** (p-value*)	623 (NS)	1028 (NS)	609 (NS)	1465 (0.013)	589 (NS)
Number (%) of CO-oriented somatic Alu	323 (51.85)	497 (48.35)	304 (49.92)	691 (47.17)	279 (47.37)
Number (%) of COUNTER-oriented somatic Alu	301 (48.15)	531 (51.65)	305 (50.08)	774 (52.83)	310 (52.63)
**Number of somatic Alu detected in 5 kB upstream gene** (p-value*)	67 (NS)	105 (NS)	61 (NS)	105 (0.021)	55 (NS)
Number (%) of CO-oriented somatic Alu	29 (43.28)	43 (40.95)	32 (52.46)	45 (42.86)	25 (45.45)
Number (%) of COUNTER-oriented somatic Alu	38 (56.72)	62 (59.05)	29 (47.54)	60 (57.14)	30 (54.55)

*—p-value based on Monte-Carlo test, 1000 permutations (see Materials and Methods for details), NS—non-significant (p>0.05)

### Validation by PCR and Sanger sequencing

**Fig 4:**
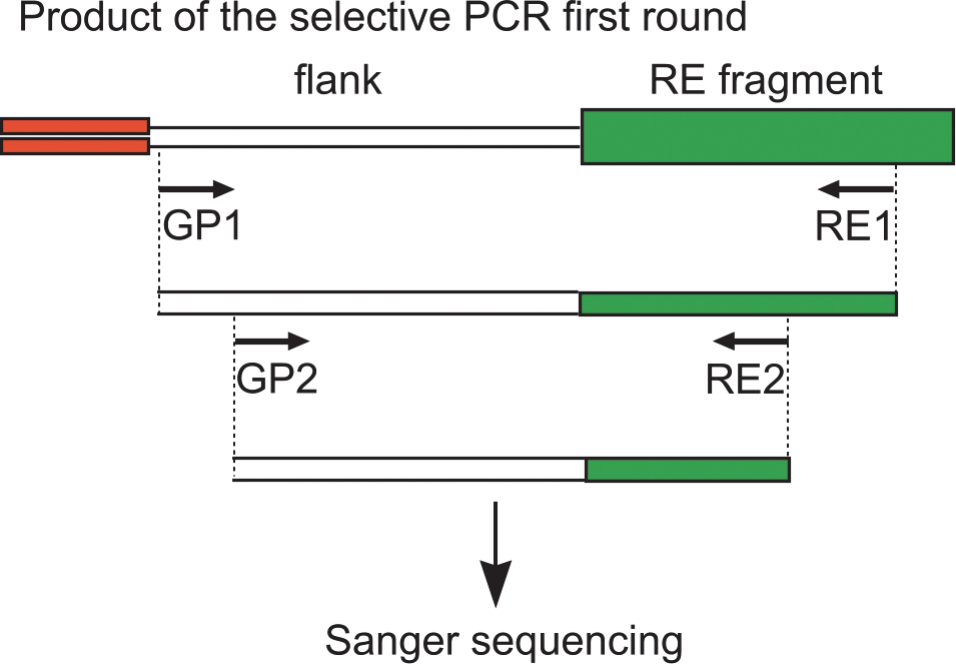
Validation of the potentially somatic retroelement insertions. Black arrows show the primers. GP primers are complementary to the flanking sequences, RE primers are complementary to the retroelement sequence (RE).

Nested PCR and Sanger sequencing of the PCR products were carried out to validate the potentially somatic insertions. We selected 34 L1 and 26 Alu elements integration events for validation (S1 Table). We confirmed the presence of the selected 15/34 L1 and 17/26 Alu element insertions in the DNA samples produced by the first step of suppression PCR and validated them as somatic (see Fig. 4 for the validation scheme). Sanger sequencing revealed single nucleotide substitutions in the sequences of several newly found REs. Comparing these RE sequences with the reference genome allowed us to identify a limited number of ancestral elements or even the exact master-copy (S1 Table). Although all of the detected somatic insertions were supposed to be represented by one or very few molecules in the initial sample, we attempted to amplify their full-length copies from the genomic DNA. We were successful in retrieving the Alu insertion previously validated in the first step of suppression PCR. However, the proper PCR product for this insertion accumulated only in one of the 12 nested reactions with 40 ng input DNA, indicating that the concentration of the template molecules in the DNA sample was very low indeed.

## Discussion

In this study we for the first time showed an increased number of L1 somatic retrotranspositions in the dentate gyrus of the human brain in comparison to other brain regions and the myocardium by directly sequencing and mapping the somatic insertions. Several recent studies proposed that activation of L1 retroelements coincides with neuronal differentiation [22, 23, 28]. Our data demonstrate that the dentate gyrus is a “hotspot” of retrotranspositional activity in the adult human brain. The increase in the number of retrotranspositions can be associated with the presence of proliferating precursor cells. Each new somatic retrotransposition could alter gene expression and hence underlie the neuron individuality. Retroelement activity in proliferating precursor cells can potentially produce subpopulations of the dentate gyrus granule cells which possess unique properties that distinguish these neurons from the neighboring ones. Considering that the adult human dentate gyrus was shown to generate around 700 neurons per day [31], a portion of neurons carrying somatic L1 retrotranspositions could be high enough to affect the local neuronal circuits.

Notably, we did not find any increase in the retrotranspositions in another putative neurogenic zone of the adult mammalian brain, the SVZ. However, several recent studies indicated that in humans, unlike rodents, the neurogenesis in the adult SVZ is negligible [33, 35]. Robust neurogenesis in the human SVZ was shown to persist for up to 18 month after birth [33]. Thus, the observed differences in the L1 retrotransposition rate between the SVZ and the DG might be associated with a significantly larger precursor cell pool in the DG in comparisons to the SVZ.

Interestingly, we showed that the number of somatic L1 retrotranspositions in promoters and genes is higher than expected for all brain samples and the myocardium. This observation is different from the data obtained by Ewing and Kazazian [38] where authors demonstrated that recent insertions are less abundant in intronic regions. This can be explained by different modes of selection acting upon the somatic and germ-line insertions. Unlike the somatic insertions, the germ-line insertions are present in every cell and can potentially affect the functioning of the whole organism. Thus, they are subject to the selection at the population level, while the brain somatic insertions are subject to completely different selective pressures. However, the uneven distribution of the discovered insertions in the genome can also result from the mapping bias: the sequencing reads better map to the unique genomic regions which comprise the actively transcribed chromatin than to the highly repetitive non-transcribed sequences.

The orientation of genic L1 insertions was observed to be biased towards counter-orientated state in all brain regions and myocardium. This can be caused by a higher negative effect of co-oriented insertions on gene expression (e.g. knock-down of gene expression) and is similar to the results obtained by Ewing and Kazazian [38]. However, Ewing and Kazazian have demonstrated the orientation bias for the insertions that established a firm foothold in the population, while our results indicate a possibility of negative selection against the co-oriented L1 insertions at the level of individual cells.

Similar to the somatic L1 insertions, the Alu insertions demonstrated the most persistent expansion in the dentate gyrus. However, the rates of Alu propagation in the rest of the samples was not as equal as for the L1. The percentage of somatic Alu elements was variable and significantly different between the regions analyzed. This can probably be explained by the differences between the mechanisms of Alu and L1 suppression [13, 39] or by the difference in the tissue-specificity of the RE expression regulating mechanisms [28, 40, 41]. Presumably, the difference in the number of detected somatic L1 and Alu insertions can result from the L1 reverse transcriptase *cis*-preference to the self RNA [42].

Interestingly, the number of somatic Alu integration events in genes and promoters was similar to the expected values for all regions analyzed, but the DG. Furthermore, in the DG while Alu integrated preferably in the genes, they avoided integrating in the promoter regions, which is opposite to L1. Moreover, DG was the only brain region in which the orientation bias of the somatic Alu insertions was observed. Altogether these facts suggest that cells of the DG carrying new Alu insertions are subject to selection.

The data on both L1 and Alu somatic insertions obtained in the current work indicate an increased retrotranspositional activity in the DG. Since our results are based on the analysis of a single donor we cannot exclude the possibility that the observed pattern of the somatic insertion distribution in brain tissues is unique and can vary in other members of the population. However, our results match the data obtained by qPCR [23] indicating that our conclusions may be generally applicable.

We have not observed any insertions which were represented by many sequencing reads on the one hand and could be considered somatic on the other. This indicates that the retroelement hops in the genome occur mainly in adult tissues or at the stage of late development, but not during embryogenesis. This corresponds to the results of Baillie and colleagues [24] who have also identified no somatic insertions represented by many reads. The vanishingly small concentrations of the somatic insertions left little chance to retrieve their sequences from the initial samples and prompted us to validate them using the first step of suppression PCR as a template. However, we managed to validate one Alu insertion in the genomic DNA, which is an exclusive case of the direct confirmation of an endogenous retroelement somatic integration.

Our results are consistent with the studies that were performed in mice. Although L1 can retrotranspose in many regions of the mouse brain [22], an injection of a lentivirus expressing L1 ORF2 fused with EGFP into the DG resulted in the L1 expression restricted to neuronal progenitor and newborn granule cells [28]. Moreover, in the hippocampi of the L1-EGFP transgenic mice, somatic L1 retrotranspositions were mainly found in cells that reside in the neurogenic niche, i.e. the SGZ of the DG [22]. Thus, L1 retrotransposition may be stimulated in proliferating progenitor cells of the mouse DG, leading to, similar to humans, an increase in the number of retrotranspositions for the DG. Since mice and humans are evolutionarily quite distant mammalian species, it is tempting to speculate that activation of L1 retrotransposition in the postnatal dentate gyrus might be an trait that is preserved in different mammals. Furthermore, since L1 retrotranspositions can be stimulated simply by running [43], activation of retrotransposition could be used by the DG as a fast response to some external (environmental) stimuli which results in the generation of neuronal pools different from the existing neurons. Taking in consideration the importance of the dentate gyrus in the formation of the hippocampal-dependent memory and learning, we can speculate that the activation of the L1 retrotransposition could have a significant effect on animal behavior. This could be addressed in the future by analyzing L1 retrotransposition in the DG of different mammalian species, and by stimulating L1 retrotransposition coupled with the analysis of animal behavior, e.g. pattern separation that was shown to be modulated by adult hippocampal neurogenesis [44, 45].

## Materials and Methods

### Contamination precautions

A three room standard was applied for sample preparation: all procedures with genomic DNA were performed in room 1; PCR preparation was performed in room 2; electrophoresis and other post-PCR activities were performed in room 3.

Primers for validating potential somatic insertions lied out of the sequences which comprised the resulting Illumina libraries in order to avoid the amplification of the contaminating molecules from these libraries.

### Tissue sources and sample preparation

Tissue samples were taken within 12 hours after death from a 72 year old male individual. Tissue samples were frozen in liquid nitrogen immediately after sectioning and then stored at -80°C. The study was approved by the local ethics committee of the Shemyakin-Ovchinnikov Institute of Bioorganic Chemistry of the Russian Academy of Sciences and conducted in accordance with the Declaration of Helsinki. As no consent could be obtained from the donor, the written consent was provided by the next of the kin. Genomic DNA was extracted from five frozen tissue samples of a single individual: cerebellum, frontal cortex, subventricular zone, dentate gyrus and myocardium, using the standard phenol-chloroform extraction method.

### Library construction and sequencing

The library construction protocol included the step of genomic DNA digestion by restriction enzymes (AluI and HaeIII for L1, AluI and RsaI for Alu), adapter ligation and two steps of suppression PCR selectively amplifying the insertion sites of the most active RE subfamilies (L1Hs and AluYa5). Sequences of the oligonucleotides used for library preparation are shown in Table 3. The libraries were sequenced on Illumina HiSeq 2000 and Illumina Genome Analyzer IIx platforms using 101 bp reads.

**Table 3:** The oligonucleotides used for the preparation of the DNA libraries.

Oligonucleotide	Sequence (5'-3')
RE-specific primers	
AY107	TCACCGTTTTAGCCGGGA
AY24	AGGCGTGAGCCACCGCGC
AY18	GAGCCACCGCGCCCGGC
3-L1HS	GAGATATACCTAATGCTAGATGACAC
3-end-L1	GCACATGTACCCTAAAACTTAGAGTA
Suppression PCR primers and adapters	
Na21st19	TGTAGCGTGAAGACGACAGAAAGGGCGTGGTGCGGAGGGCGGT
st20	ACCGCCCTCC
Na15Na21	AGCAGCGAACTCAGTACAACATGTAGCGTGAAGACGACAGAA
Na15	AGCAGCGAACTCAGTACAACA
st19	AGGGCGTGGTGCGGAGGGCGGT

### Sequence mapping and analysis

Data analysis included the use of standard tools: Bowtie2 [46, 47] and Galaxy [48–50], Perl and Python scripts. The raw data processing protocol included mapping the reads to the reference genome (UCSC hg19), identifying the coordinates of RE insertions, retrieving the coordinates of potentially somatic insertions, and several steps of filtering out false-positive results.

### Analysis of the distribution of potential somatic insertions in genome

The coordinates of the potential somatic insertions were intersected with the coordinates of the known genes and genes promoter regions (5,000 bp upstream of the genes transcription start sites) present in hg19 by the Galaxy tool “Join”. Statistical analysis was done using R software [51] including qcc package [52].

### Statistical data analysis

The analysis of Alu and L1 distributions in different brain areas and of the genomic distribution of the somatic L1 and Alu insertions was performed using an overdispersion test. The distribution of Alu and L1 in the dentate gyrus was compared with the distribution in all other samples combined using the Poisson test. Binomial tests were applied for the analysis of somatic L1 and Alu orientation relative to nearby genes. Monte Carlo simulations of random retroelement distributions throughout the genome were performed to analyze the randomness of the Alu and L1 distributions in promoters and genes.

### Validation of the somatic insertions

Nested PCR was performed for the validation of the selected somatic retroelement insertions. PCR products were Sanger sequenced. Primer structures and sequences are given in S1 Table.

### PCR amplification of somatic insertions from genomic DNA

Nested PCR was performed to amplify the RE insertion flanking sequences from gDNA. Primer structures are given in S1 Table.

For more details see **S1 Materials and Methods**

### Accession Numbers

The European Nucleotide Archive accession number for the Illumina sequences reported in this paper is PRJEB4787.

## Supporting Information

S1 Table The L1 and Alu insertions chosen for confirmation by PCR and Sanger sequencing, primers used for validation and Sanger sequences. (XLSX)

S2 Table Power analysis for statistical tests. (PDF)

S1 Materials and Methods Supplementary materials and methods. (PDF)

## References

[1] Bannert N, Kurth R (2004) Retroelements and the human genome: new perspectives on an old relation. Proceedings of the National Academy of Sciences of the United States of America 101 Suppl 2: 14572–14579. 15310846 10.1073/pnas.0404838101 PMC521986

[2] Feng Q, Moran JV, Kazazian HH Jr, Boeke JD (1996) Human L1 retrotransposon encodes a conserved endonuclease required for retrotransposition. Cell 87: 905–916. 8945517 10.1016/s0092-8674(00)81997-2

[3] Batzer MA, Deininger PL (2002) Alu repeats and human genomic diversity. Nat Rev Genet 3: 370–379. 11988762 10.1038/nrg798

[4] Amosova AL, Komkov A, Ustiugova SV, Mamedov IZ, Lebedev Iu B (2009) [Retroposons in modern human genome evolution]. Bioorg Khim 35: 779–788. 20208577 10.1134/s1068162009060053

[5] Lebedev YB, Amosova AL, Mamedov IZ, Fisunov GY, Sverdlov ED (2007) Most recent AluY insertions in human gene introns reduce the content of the primary transcripts in a cell type specific manner. Gene 390: 122–129. 17118582 10.1016/j.gene.2006.09.031

[6] Polak P, Domany E (2006) Alu elements contain many binding sites for transcription factors and may play a role in regulation of developmental processes. BMC genomics 7: 133 16740159 10.1186/1471-2164-7-133 PMC1513395

[7] Belancio VP, Hedges DJ, Deininger P (2006) LINE-1 RNA splicing and influences on mammalian gene expression. Nucleic acids research 34: 1512–1521. 16554555 10.1093/nar/gkl027 PMC1415225

[8] Deininger PL, Batzer MA (1999) Alu repeats and human disease. Mol Genet Metab 67: 183–193. 10381326 10.1006/mgme.1999.2864

[9] Callinan PA, Batzer MA (2006) Retrotransposable elements and human disease. Genome Dyn 1: 104–115. 10.1159/000092503 18724056

[10] Yang N, Kazazian HH Jr., (2006) L1 retrotransposition is suppressed by endogenously encoded small interfering RNAs in human cultured cells. Nat Struct Mol Biol 13: 763–771. 16936727 10.1038/nsmb1141

[11] Smalheiser NR, Torvik VI (2006) Alu elements within human mRNAs are probable microRNA targets. Trends Genet 22: 532–536. 16914224 10.1016/j.tig.2006.08.007

[12] Aravin AA, Hannon GJ, Brennecke J (2007) The Piwi-piRNA pathway provides an adaptive defense in the transposon arms race. Science 318: 761–764. 17975059 10.1126/science.1146484

[13] Muotri AR, Marchetto MC, Coufal NG, Oefner R, Yeo G, et al (2010) L1 retrotransposition in neurons is modulated by MeCP2. Nature 468: 443–446. 10.1038/nature09544 21085180 PMC3059197

[14] Reilly MT, Faulkner GJ, Dubnau J, Ponomarev I, Gage FH (2013) The role of transposable elements in health and diseases of the central nervous system. The Journal of neuroscience: the official journal of the Society for Neuroscience 33: 17577–17586. 10.1523/JNEUROSCI.3369-13.2013 24198348 PMC3818539

[15] Erwin JA, Marchetto MC, Gage FH (2014) Mobile DNA elements in the generation of diversity and complexity in the brain. Nature reviews Neuroscience 15: 497–506. 10.1038/nrn3730 25005482 PMC4443810

[16] Iskow RC, McCabe MT, Mills RE, Torene S, Pittard WS, et al (2010) Natural mutagenesis of human genomes by endogenous retrotransposons. Cell 141: 1253–1261. 10.1016/j.cell.2010.05.020 20603005 PMC2943760

[17] Lee E, Iskow R, Yang L, Gokcumen O, Haseley P, et al (2012) Landscape of somatic retrotransposition in human cancers. Science 337: 967–971. 10.1126/science.1222077 22745252 PMC3656569

[18] Solyom S, Ewing AD, Rahrmann EP, Doucet T, Nelson HH, et al (2012) Extensive somatic L1 retrotransposition in colorectal tumors. Genome research 22: 2328–2338. 10.1101/gr.145235.112 22968929 PMC3514663

[19] Shukla R, Upton KR, Munoz-Lopez M, Gerhardt DJ, Fisher ME, et al (2013) Endogenous retrotransposition activates oncogenic pathways in hepatocellular carcinoma. Cell 153: 101–111. 10.1016/j.cell.2013.02.032 23540693 PMC3898742

[20] Garcia-Perez JL, Marchetto MC, Muotri AR, Coufal NG, Gage FH, et al (2007) LINE-1 retrotransposition in human embryonic stem cells. Hum Mol Genet 16: 1569–1577. 17468180 10.1093/hmg/ddm105

[21] Kano H, Godoy I, Courtney C, Vetter MR, Gerton GL, et al (2009) L1 retrotransposition occurs mainly in embryogenesis and creates somatic mosaicism. Genes Dev 23: 1303–1312. 10.1101/gad.1803909 19487571 PMC2701581

[22] Muotri AR, Chu VT, Marchetto MC, Deng W, Moran JV, et al (2005) Somatic mosaicism in neuronal precursor cells mediated by L1 retrotransposition. Nature 435: 903–910. 15959507 10.1038/nature03663

[23] Coufal NG, Garcia-Perez JL, Peng GE, Yeo GW, Mu Y, et al (2009) L1 retrotransposition in human neural progenitor cells. Nature 460: 1127–1131. 10.1038/nature08248 19657334 PMC2909034

[24] Baillie JK, Barnett MW, Upton KR, Gerhardt DJ, Richmond TA, et al (2011) Somatic retrotransposition alters the genetic landscape of the human brain. Nature 479: 534–537. 10.1038/nature10531 22037309 PMC3224101

[25] Arokium H, Kamata M, Kim S, Kim N, Liang M, et al (2014) Deep Sequencing Reveals Low Incidence of Endogenous LINE-1 Retrotransposition in Human Induced Pluripotent Stem Cells. PloS one 9: e108682 10.1371/journal.pone.0108682 25289675 PMC4188539

[26] Evrony GD, Cai X, Lee E, Hills LB, Elhosary PC, et al (2012) Single-neuron sequencing analysis of l1 retrotransposition and somatic mutation in the human brain. Cell 151: 483–496. 10.1016/j.cell.2012.09.035 23101622 PMC3567441

[27] Bundo M, Toyoshima M, Okada Y, Akamatsu W, Ueda J, et al (2014) Increased l1 retrotransposition in the neuronal genome in schizophrenia. Neuron 81: 306–313. 10.1016/j.neuron.2013.10.053 24389010

[28] Kuwabara T, Hsieh J, Muotri A, Yeo G, Warashina M, et al (2009) Wnt-mediated activation of NeuroD1 and retro-elements during adult neurogenesis. Nat Neurosci 12: 1097–1105. 10.1038/nn.2360 19701198 PMC2764260

[29] Zhao C, Deng W, Gage FH (2008) Mechanisms and functional implications of adult neurogenesis. Cell 132: 645–660. 10.1016/j.cell.2008.01.033 18295581

[30] Eriksson PS, Perfilieva E, Bjork-Eriksson T, Alborn AM, Nordborg C, et al (1998) Neurogenesis in the adult human hippocampus. Nature medicine 4: 1313–1317. 9809557 10.1038/3305

[31] Spalding KL, Bergmann O, Alkass K, Bernard S, Salehpour M, et al (2013) Dynamics of hippocampal neurogenesis in adult humans. Cell 153: 1219–1227. 10.1016/j.cell.2013.05.002 23746839 PMC4394608

[32] Sanai N, Tramontin AD, Quinones-Hinojosa A, Barbaro NM, Gupta N, et al (2004) Unique astrocyte ribbon in adult human brain contains neural stem cells but lacks chain migration. Nature 427: 740–744. 14973487 10.1038/nature02301

[33] Sanai N, Nguyen T, Ihrie RA, Mirzadeh Z, Tsai HH, et al (2011) Corridors of migrating neurons in the human brain and their decline during infancy. Nature 478: 382–386. 10.1038/nature10487 21964341 PMC3197903

[34] Curtis MA, Kam M, Nannmark U, Anderson MF, Axell MZ, et al (2007) Human neuroblasts migrate to the olfactory bulb via a lateral ventricular extension. Science 315: 1243–1249. 17303719 10.1126/science.1136281

[35] Wang C, Liu F, Liu YY, Zhao CH, You Y, et al (2011) Identification and characterization of neuroblasts in the subventricular zone and rostral migratory stream of the adult human brain. Cell research 21: 1534–1550. 10.1038/cr.2011.83 21577236 PMC3365638

[36] Mamedov I, Batrak A, Buzdin A, Arzumanyan E, Lebedev Y, et al (2002) Genome-wide comparison of differences in the integration sites of interspersed repeats between closely related genomes. Nucleic acids research 30: e71 12136119 10.1093/nar/gnf071 PMC135772

[37] Mamedov IZ, Arzumanyan ES, Amosova AL, Lebedev YB, Sverdlov ED (2005) Whole-genome experimental identification of insertion/deletion polymorphisms of interspersed repeats by a new general approach. Nucleic acids research 33: e16 15673711 10.1093/nar/gni018 PMC548376

[38] Ewing AD, Kazazian HH Jr., (2010) High-throughput sequencing reveals extensive variation in human-specific L1 content in individual human genomes. Genome research 20: 1262–1270. 10.1101/gr.106419.110 20488934 PMC2928504

[39] Khatua AK, Taylor HE, Hildreth JE, Popik W (2010) Inhibition of LINE-1 and Alu retrotransposition by exosomes encapsidating APOBEC3G and APOBEC3F. Virology 400: 68–75. 10.1016/j.virol.2010.01.021 20153011 PMC2851184

[40] Carmell MA, Girard A, van de Kant HJ, Bourc'his D, Bestor TH, et al (2007) MIWI2 is essential for spermatogenesis and repression of transposons in the mouse male germline. Developmental cell 12: 503–514. 17395546 10.1016/j.devcel.2007.03.001

[41] Aravin AA, Sachidanandam R, Girard A, Fejes-Toth K, Hannon GJ (2007) Developmentally regulated piRNA clusters implicate MILI in transposon control. Science 316: 744–747. 17446352 10.1126/science.1142612

[42] Wei W, Gilbert N, Ooi SL, Lawler JF, Ostertag EM, et al (2001) Human L1 retrotransposition: cis preference versus trans complementation. Molecular and cellular biology 21: 1429–1439. 11158327 10.1128/MCB.21.4.1429-1439.2001 PMC99594

[43] Muotri AR, Zhao C, Marchetto MC, Gage FH (2009) Environmental influence on L1 retrotransposons in the adult hippocampus. Hippocampus 19: 1002–1007. 10.1002/hipo.20564 19771587 PMC2758700

[44] Clelland CD, Choi M, Romberg C, Clemenson GD Jr., Fragniere A, et al (2009) A functional role for adult hippocampal neurogenesis in spatial pattern separation. Science 325: 210–213. 10.1126/science.1173215 19590004 PMC2997634

[45] Nakashiba T, Cushman JD, Pelkey KA, Renaudineau S, Buhl DL, et al (2012) Young dentate granule cells mediate pattern separation, whereas old granule cells facilitate pattern completion. Cell 149: 188–201. 10.1016/j.cell.2012.01.046 22365813 PMC3319279

[46] Langmead B, Trapnell C, Pop M, Salzberg SL (2009) Ultrafast and memory-efficient alignment of short DNA sequences to the human genome. Genome Biol 10: R25 10.1186/gb-2009-10-3-r25 19261174 PMC2690996

[47] Langmead B, Salzberg SL (2012) Fast gapped-read alignment with Bowtie 2. Nat Methods 9: 357–359. 10.1038/nmeth.1923 22388286 PMC3322381

[48] Giardine B, Riemer C, Hardison RC, Burhans R, Elnitski L, et al (2005) Galaxy: a platform for interactive large-scale genome analysis. Genome research 15: 1451–1455. 16169926 10.1101/gr.4086505 PMC1240089

[49] Blankenberg D, Von Kuster G, Coraor N, Ananda G, Lazarus R, et al. (2010) Galaxy: a web-based genome analysis tool for experimentalists. Curr Protoc Mol Biol Chapter 19: Unit 19 10 11–21. 10.1002/0471142727.mb1910s89 PMC4264107 20069535

[50] Goecks J, Nekrutenko A, Taylor J (2010) Galaxy: a comprehensive approach for supporting accessible, reproducible, and transparent computational research in the life sciences. Genome Biol 11: R86 10.1186/gb-2010-11-8-r86 20738864 PMC2945788

[51] R Core Team (2014) R: A language and environment for statistical computing. Vienna, Austria: R Foundation for Statistical Computing 10.1016/j.jcis.2014.12.029

[52] Scrucca L (2004) qcc: an R package for quality control charting and statistical process control. R News 4/1: 22–27.

