# Supplementary material for: The Evidence for Increased L1 Activity in the Site of Human Adult Brain Neurogenesis
Source: PLoS One. 2015 Feb 17;10(2):e0117854. doi: 10.1371/journal.pone.0117854 (PMC4331437; doi:10.1371/journal.pone.0117854)
Supplement: S2 Table — (PDF) [file pone.0117854.s002.pdf]

Table S2. Power analysis

RE type	Alu		L1									
Genomic region	genes	5 kB upstream genes	genes					5 kB upstream genes				
Tissue	DG	DG	CB	FC	SVZ	DG	MY	CB	FC	SVZ	DG	MY
Power, %	76	64	95	56	93	98	71	80	74	95	98	52
Difference required for 80% power	69	28	N/A	28	N/A	N/A	43	N/A	13	N/A	N/A	21

The power of the test was calculated for each comparison and the power correlated with the difference and the p value (the power is shown only for $p < 0.05$). Since for several comparisons the power was < 80%, we also calculated how large the difference between simulated and experimental samples should be to obtain the power = 80%. DG - dentate gyrus, CB - cerebellum, FC - frontal cortex, SVZ - subventricular zone, MY – myocardium, N/A – not applicable
