# Supplementary material for: The Evidence for Increased L1 Activity in the Site of Human Adult Brain Neurogenesis
Source: PLoS One. 2015 Feb 17;10(2):e0117854. doi: 10.1371/journal.pone.0117854 (PMC4331437; doi:10.1371/journal.pone.0117854)
Supplement: S1 Materials and Methods — (PDF) [file pone.0117854.s003.pdf]

SUPPLEMENTARY MATERIALS AND METHODS

Tissue sources and sample preparation

The tissue specimen (100-200 mg) was homogenized in liquid nitrogen and incubated in 3 ml of lysis buffer (60 mM Tris, 100 мM EDTA, 0.5 % SDS) with 0.4 mg RNAse A (Qiagen, USA) at 37°C for 1 hour then 1.5 mg of Proteinase K (Qiagen, USA) was added and incubated overnight at 50°C. DNA was extracted by a phenol-chloroform method. An equal volume of phenol (pH 8.0) was added to the tissue lysate, mixed gently for 30 min and centrifuged for 15 min at 4,500 *g*. The aqueous phase was collected, gently mixed with an equal volume of phenol-chloroform (1:1) and centrifuged. The aqueous phase was collected, gently mixed with an equal volume of chloroform and centrifuged. Genomic DNA was precipitated from the aqueous phase by ethanol.

Library construction and sequencing

Genomic DNA was digested by restriction enzymes AluI and RsaI (Fermentas, Lithuania) or AluI (Fermentas, Lithuania) and HaeIII (NEB, USA) for preparing Alu or L1 libraries respectively. Genomic DNA (2 μg) was incubated for 12 hours with 20 U of the enzyme in the Y Tango buffer (Fermentas, Lithuania) for Fermentas enzymes or in the NEBuffer 4 (NEB, USA) for HaeIII. Restriction products were purified using the QIAquick PCR Purification Kit (Qiagen, USA).

Adapter oligonucleotides were ligated to the restriction fragments. The adapter oligonucleotide Na21st19 was preliminarily hybridized to the oligonucleotide st20 (see Table 3 for oligonucleotide sequences). The hybridization mixture of 4 μl contained oligonucleotides (5 μM each), 10 mM Tris-HCl, 10 mM $MgCl_2$. The mixture was incubated for 5 min at 68°C and then cooled down by 0.1^{o}C/sec to 15°C. The 10 μl ligation mixture contained 120 pmoles of Na21st19/st20 duplex, 6 U of T4 DNA ligase in a T4 reaction buffer (Promega, USA) and 2 μg of the restricted genomic DNA. The reaction was carried out overnight at +16°C.

DNA amplification for library preparation was performed in two subsequent suppression PCR steps. See Table 3 for structures of all oligonucleotides used. The first step 25 μl PCR reaction contained 1/50 of the total amount of ligation products, 0.4 μM retroelement-specific primer (AY107 for Alu and 3-L1HS for L1 library preparation), 0.16 μM Na15 primer, 0.02 μM Na15Na21 primer, dNTP (0.125 μM each), 1 U of Encyclo polymerase (Evrogen, Russia) in the reaction buffer. The amplification profile for Alu libraries was as follows: initial end extension for 4 min at 72°C, followed by 13 or 16 cycles (for Alu and L1 libraries respectively) of 20 sec at 94°C, 15 sec at 65°C, 1 min at 72°C and a final elongation for 2 min at 72°C. Fifty identical

reactions were performed for library preparation. PCR products were combined, purified with the QIAquick PCR Purification Kit and concentrated to the volume of 120 μl.

The second step 25 μl PCR reaction contained 1/1200 of the combined first step products, 0.4 μM retroelement-specific primer (AY24 or AY18 for Alu and 3-end-L1 for L1 library preparation), 0.16 μM st19 primer, dNTP (0.125 μM each), 1 U of Encyclo polymerase (Evrogen, Russia) in the reaction buffer. The amplification profile for Alu library preparation was as follows: 10 cycles of 20 sec at 94°C, 15 sec at 68°C, 1 min at 72°C and a final extension for 2 min at 72°C. The amplification profile for L1 library preparation was: 12 cycles of 20 sec at 94°C, 15 sec at 65°C, 1 min at 72°C and a final extension for 2 min at 72°C. Thirty identical reactions were performed for library preparation. PCR products were combined, purified with the QIAquick PCR Purification Kit and concentrated to the volume of 60 μl.

DNA concentration was measured by Qubit 2.0. Five hundred ng of DNA from each library obtained with one of the restriction enzymes (which comprises 1/5 of the total amount of DNA in a produced sample) was taken for sequencing.

Sequence mapping and analysis

The analysis of Alu libraries reads consisted of the following steps:

1. Extraction of reads which contained an Alu fragment.
2. Low quality base trimming.
3. Anti-chimeric filter 1: removing reads containing restriction sites (potentially chimeric reads).
4. Trimming of the Alu fragment from the 5'-end of the reads and pair-end mapping to the reference human genome was performed by Bowtie2. Settings different from default were: -p 8 -X 600 -k 2 --no-mixed --no-discordant.
5. Extraction of the unambiguously mapped reads from the Bowtie2 output files.
6. Building tables of coordinates and merging coordinates into peaks.
7. Anti-chimeric filter 2: Removing coordinates which have a restriction site located within 50 bp towards the flanking region of the insertion.
8. Matching the obtained coordinates with the coordinates of the known Alu and L1 present in hg19 and databases of polymorphic retroelements (dbRIP and PRED [1, 2]) by the Galaxy tool "Join".

The analysis of L1 libraries reads consisted of the following steps:

1. Extraction of reads which contained an L1 fragment.
2. Low quality base trimming.
3. Anti-chimeric filter 1: removing reads containing restriction sites (potentially chimeric reads).
4. Each of the mate-paired L1 libraries' files was split into 3. Reads which had a fragment of an informative genomic sequence in mate 1 (a fragment was considered informative if represented a non-LINE, non-polyA sequence and was at least 25 nt long) were extracted to the first pair of files (type 1 reads). Reads which had no informative fragment in mate 1, but had a stretch of at least 4 thymine nucleotides at the 3'-end of the mate 2 (which could represent the 3'-end nucleotides of the LINE polyA-tail) were extracted to the second pair of files (type 2 reads). Reads which had neither an informative fragment in mate 1 nor a LINE polyA-tail at the 3'-end of the mate 2 were excluded from further analysis. Type 1 reads were further processed as follows: the LINE fragment and the adjacent polyA tail were trimmed from the 5'-end of the mate 1. Pair-end mapping to UCSC hg19 reference genome was performed by Bowtie2. Settings different from default were: -p 8 -X 600 -k 2 --no-mixed --no-discordant. Type 2 reads were further processed as follows: the LINE polyA tail was trimmed from the 3'-end of the mate 2. Mate 2 single-end was mapped to UCSC hg19 by Bowtie2. Settings different from default were: -p 8 --mp 12,8
5. Extraction of the coordinates of the unambiguously mapped concordant reads (for type 1 libraries) and unambiguously mapped reads (for type 2 libraries) from the Bowtie2 output files.
6. Building tables of coordinates and merging coordinates into peaks
7. Anti-chimeric filter 2: Removing coordinates which have a restriction site located within 50 bp towards the flanking region of the insertion
8. Matching the obtained coordinates with the coordinates of the known L1 present in hg19 by the Galaxy tool "Join".

Statistical data analysis

For the analysis of Alu and L1 distributions in different brain areas, we employed an overdispersion test using the binomial distribution to analyze whether all samples are equal or not. Poisson test was used to compare the distribution of Alu and L1 in the dentate gyrus with all other samples combined. Subsequently, we used Poisson tests for pair-wise comparisons between the samples.

For the analysis of the genomic distribution of the somatic L1 and Alu insertions, i.e. in genes, 5 kb regions upstream the genes or in all other regions, we used an overdispersion test, similarly to the analysis described above.

For the analysis of somatic L1 and Alu orientation relative to nearby genes (for those retroelements integrated into introns or 5 kb regions upstream genes only), we used binomial tests to check the null-hypothesis of the co- and counter- oriented Alu- and L1 being equifrequent. To study differences in retroelement orientation relative to nearby genes across the brain regions and myocardium, mean values for co- and counter-oriented L1 or Alu were used and analyzed by an overdispersion test.

For analyzing the randomness of the Alu and L1 distributions in promoters and genes, Monte Carlo simulations of random retroelement distributions throughout the genome were performed 1000 times and compared to the values obtained from sequencing analysis. For each sample the number of random coordinates equal to the number of somatic insertions in the sample was generated using the hg19 genome assembly (excluding telomeric, centromeric and other N-base regions) and intersected with a list of gene and promoter RefSeq coordinates. P-values were then produced by comparing the number of retroelements inserted in genes or promoters to the quantiles of the Monte Carlo distribution. To calculate the power of the analysis, we performed another 1,000 simulations, but now the distribution of Alu and L1 (i.e. the probability for LINE or Alu to be inserted into gene or promoter region) was adjusted to the data obtained from the sequencing results. We then calculated the percentage of the adjusted simulations that were significantly different from the initial 1,000 random simulations. We found that a difference of 15-30 and 30-70 retroelements (depending on the sample size) was required to achieve a power of 80% for promoter and gene regions, respectively, which was well within the range of our observed values. The data are summarized in the Table S2

Validation of the somatic insertions

Nested PCR was performed for the validation of the chosen somatic retroelement insertions. Products of the first suppression PCR were taken as a template for the first reaction. The 25 µl PCR reaction contained 1 µl template DNA, 0.4 µM retroelement-specific primer (RE outer), 0.4 µM Flank outer primer, dNTPs (0.125 µM each), 1 U of HS Taq polymerase (Evrogen, Russia) in the reaction buffer. The amplification profile was as follows: preheating for 5 min at 95°C, 22 cycles of 20 sec at 94°C, 20 sec at 63°C, 40 sec at 72°C. Products of this reaction were diluted 100 times and taken for the nested PCR. The 25 µl PCR reaction contained 1 µl template DNA, 0.4 µM retroelement-specific primer (RE inner), 0.4 µM Flank inner primer, dNTP (0.125 µM each), 1 U of HS Taq polymerase (Evrogen, Russia) in the reaction buffer. The amplification profile was as follows: preheating for 5 min at 95°C, 28 cycles of 20 sec at 94°C, 20 sec at 63°C, 40 sec at 72°C. The obtained PCR products were sequenced (Evrogen, Russia) by the ABI PRISM® 3100 Genetic Analyzer. Primer structures are given in Table S1. PCR products were Sanger sequenced (sequences are shown in Table S1).

PCR amplification of somatic insertions from genomic DNA

Nested PCR was performed to attempt amplification of RE insertions' flanking sequences from gDNA. The first 25 µl PCR reaction for Alu contained 40 ng template DNA, 0.4 µM Flank outer for primer, 0.4 µM Flank outer rev primer, dNTPs (0.125 µM each), 1 U of Encyclo polymerase (Evrogen, Russia) in the reaction buffer. The first 25 µl PCR reaction for L1 contained 40 ng template DNA, 0.4 µM RE outer for primer, 0.4 µM Flank outer rev primer, dNTP (0.125 µM each), 1 U of Encyclo polymerase (Evrogen, Russia) in the reaction buffer. The amplification profile for the reaction was: 25 cycles of 20 sec at 94°C, 20 sec at 63°C, 40 sec at 72°C. From 3 to 12 such reactions were performed with each gDNA template. Products of the first reaction were diluted 100 times and taken for two nested PCRs. Then two nested reactions were performed for Alu. Twenty five µl nested reaction 1 contained 1 µl template DNA, 0.4 µM Flank inner for primer, 0.4 µM RE inner rev primer, dNTP (0.125 µM each), 1 U of Encyclo polymerase (Evrogen, Russia). Twenty five µl nested reaction 2 contained 1 µl template DNA, 0.4 µM RE inner for primer, 0.4 µM Flank inner primer, dNTP (0.125 µM each), 1 U of Encyclo polymerase (Evrogen, Russia). One nested reaction was performed for L1. Twenty five µl reaction contained 1 µl template DNA, 0.4 µM RE inner for primer, 0.4 µM Flank inner rev primer, dNTP (0.125 µM each), 1 U of Encyclo polymerase (Evrogen, Russia). The amplification profile for the reactions was: 30 cycles of 20 sec at 94°C, 20 sec at 63°C, 40 sec at 72°C. Primer structures are given in Table S1.

Supplementary References

1. Wang J, Song L, Grover D, Azrak S, Batzer MA, et al. (2006) dbRIP: a highly integrated database of retrotransposon insertion polymorphisms in humans. Human mutation 27: 323-329.
2. Mamedov IZ, Amosova AL, Fisunov G and Lebedev Iu B (2008) [A new database on polymorphic retroelements in human genome (PRED)]. Molekuliarnaia biologiia 42: 721-727.
